# Crystal structure and Hirshfeld surface analysis of (2*Z*)-4-oxo-4-{phen­yl[(2*E*)-3-phenyl­prop-2-en-1-yl]amino}­but-2-enoic acid

**DOI:** 10.1107/S2056989025010746

**Published:** 2026-01-01

**Authors:** Kseniia A. Alekseeva, Alexandra G. Kutasevich, Anna A. Zhernosek, Mehmet Akkurt, Gizachew Mulugeta Manahelohe, Punhan J. Jamalov, Khudayar I. Hasanov

**Affiliations:** a RUDN University, 6 Miklukho-Maklaya St., Moscow 117198, Russian Federation; b Department of Physics, Faculty of Sciences, Erciyes University, 38039 Kayseri, Türkiye; c Department of Chemistry, University of Gondar, PO Box 196, Gondar, Ethiopia; d Department of Chemical Engineering, Baku Engineering University, Hasan str. 120, Baku, Absheron AZ0101, Azerbaijan; e Azerbaijan Medical University, Scientific Research Centre (SRC), A. Kasumzade St. 14, AZ 1022, Baku, Azerbaijan; Katholieke Universiteit Leuven, Belgium

**Keywords:** crystal structure, hydrogen bonds, dimers, Hirshfeld surface analysis

## Abstract

In the crystal structure of the title compound, C_19_H_17_NO_3_, C—H⋯O hydrogen bonds connect mol­ecular pairs to produce dimers with an *R*^2^_2_(16) ring motif. Additionally, C—H⋯π inter­actions form ribbons along the [101] direction. Van der Waals inter­actions between the ribbons help to consolidate the mol­ecular packing. Hirshfeld surface analysis shows that H⋯H (45.5%), C⋯H/H⋯C (30.4%), and O⋯H/H⋯O (19.3%) inter­actions are the main contributors to the crystal packing.

## Abstract 2

In the crystal, C—H⋯O hydrogen bonds link the mol­ecular pairs to form dimers with an *R*^2^_2_(16) ring motif. Additionally, ribbons are formed along the [101] direction by C—H⋯π inter­actions. van der Waals inter­actions between the ribbons contribute to the cohesion of the mol­ecular packing.

## 1. Chemical context

The intra­molecular Diels–Alder (IMDA) reaction provides an efficient and versatile approach for the one-step construction of condensed carbo- and heterocyclic systems (Krishna *et al.*, 2022 ▸). However, successful implementation of this approach requires the presence of both diene and dienophile fragments within the same mol­ecule, which is not always easily achievable. The starting compounds suitable for the intra­molecular Diels–Alder reaction often possess complex mol­ecular architectures and are obtained through multistep synthetic sequences (Patre *et al.*, 2007 ▸; Hu *et al.*, 2018 ▸).
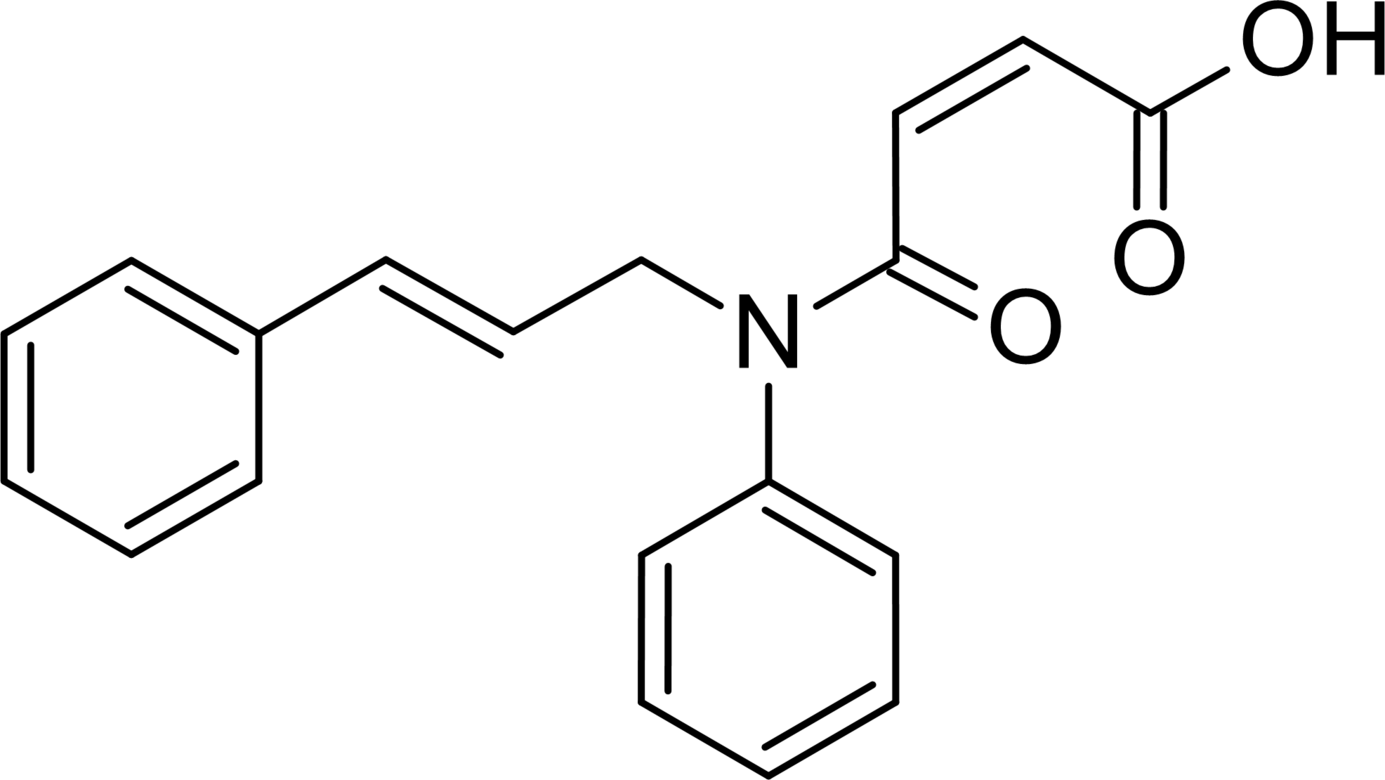


The title compound can be synthesized in a single, straightforward step from amine, which, in turn, is readily prepared by the condensation of cinnamaldehyde with aniline followed by reduction of the resulting C=N bond. In the title compound, the vinyl arene fragment acts as a diene, while the male­imide fragment serves as a dienophile, making it a promising substrate for investigation of the intra­molecular Diels–Alder reaction. It should be noted here, that the heterocyclic products derived from the title compound and its substituted analogues have demonstrated notable anti­viral activity against the H1N1 influenza virus (Voronov *et al.*, 2018 ▸). Moreover, the carb­oxy­lic group in the title compound can be used in the synthesis of metal complex catalysts (Aliyeva *et al.*, 2024 ▸; Huseynov *et al.*, 2018 ▸, 2021 ▸), or act as the hydrogen-bond donor/acceptor in the synthesis of new supra­molecular compounds (Burkin *et al.*, 2024 ▸; Maharramov *et al.*, 2011 ▸).

## 2. Structural commentary

**Figure 1:**
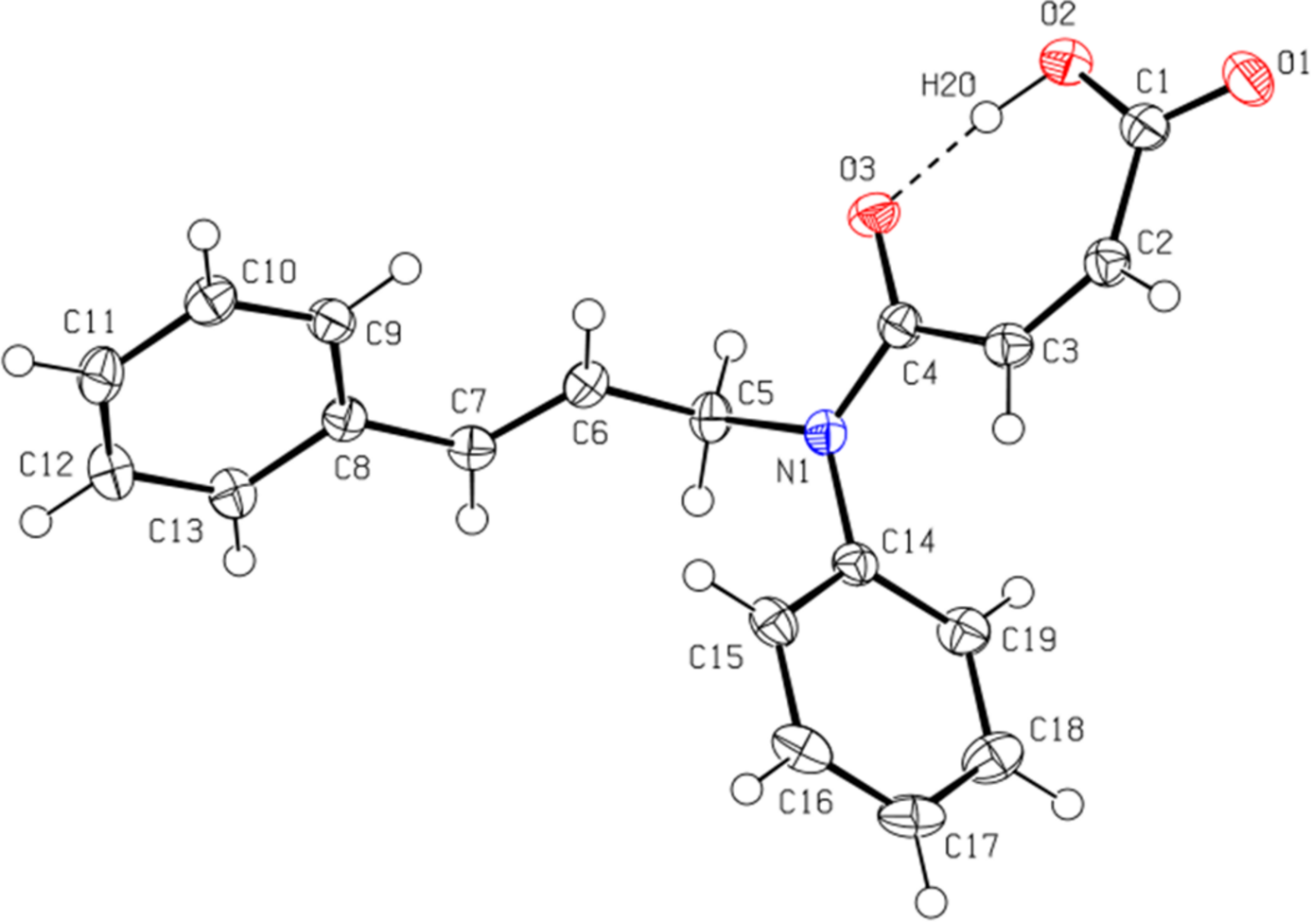
The mol­ecular structure of the title compound, showing the atom labelling and displacement ellipsoids drawn at the 50% probability level. The intra­molecular O—H⋯O hydrogen bond (dashed line) forms an *S*(7) motif between the hydroxyl hydrogen and the carbonyl oxygen atom.

The title compound (Fig. 1 ▸) exhibits a *Z* configuration about the C2=C3 double bond and *E* configuration about the C6=C7 double bond. The mol­ecular conformation is consolidated by an intra­molecular O—H⋯O hydrogen bond forming an *S*(7) motif (Table 1 ▸, Fig. 1 ▸; Bernstein *et al.*, 1995 ▸). The C—N bond lengths are: C4—N1 = 1.3544 (19), C5—N1 = 1.488 (2), and C14—N1 = 1.447 (2) Å. The sum of the angles around the N-atom [C4—N1—C14 = 123.00 (13), C4—N1—C5 = 118.99 (13), and C14—N1—C5 = 117.96 (12)°] is 359.95 (13)°. The mol­ecular conformation is roughly planar [maximum deviations: −1.611 (2) Å for C5, 1.316 (2) Å for C10, −1.026 (2) Å for C7, and 1.189 (2) Å for C16]. The angle between the phenyl rings is 68.01 (8)°. The torsion angles C6—C7—C8—C9, C5—C6—C7—C8, N1—C5—C6—C7, C5—N1—C14—C15, C5—N1—C4—O3, C5—N1—C4—C3, N1—C4—C3—C2, C4—C3—C2—C1, C3—C2—C1—O1, and C3—C2—C1—O2 are −3.2 (3), −178.36 (14), −130.63 (16), −73.02 (17), −0.4 (2), 176.76 (12), 170.26 (15), −1.5 (3), −173.25 (16) and 7.5 (3)°, respectively.

## 3. Supra­molecular features and Hirshfeld surface analysis

**Figure 2:**
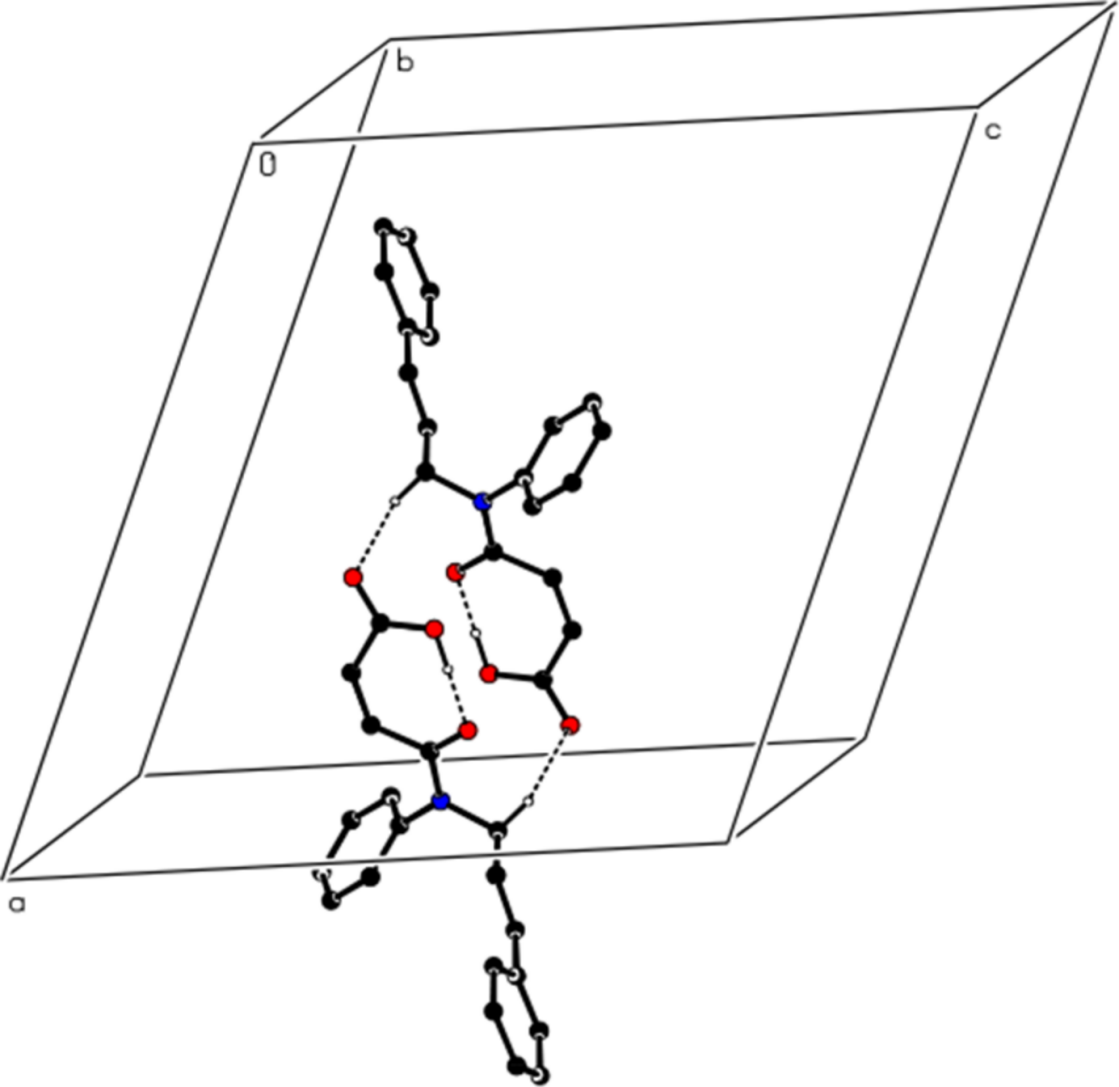
A partial view of the intra­molecular O—H⋯O hydrogen bonds forming an *S*(7) motif and the inter­molecular C—H⋯O hydrogen bonds between mol­ecular pairs forming an 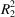
(16) motif. Hydrogen atoms not involved in hydrogen bonding have been omitted for clarity.

**Figure 4:**
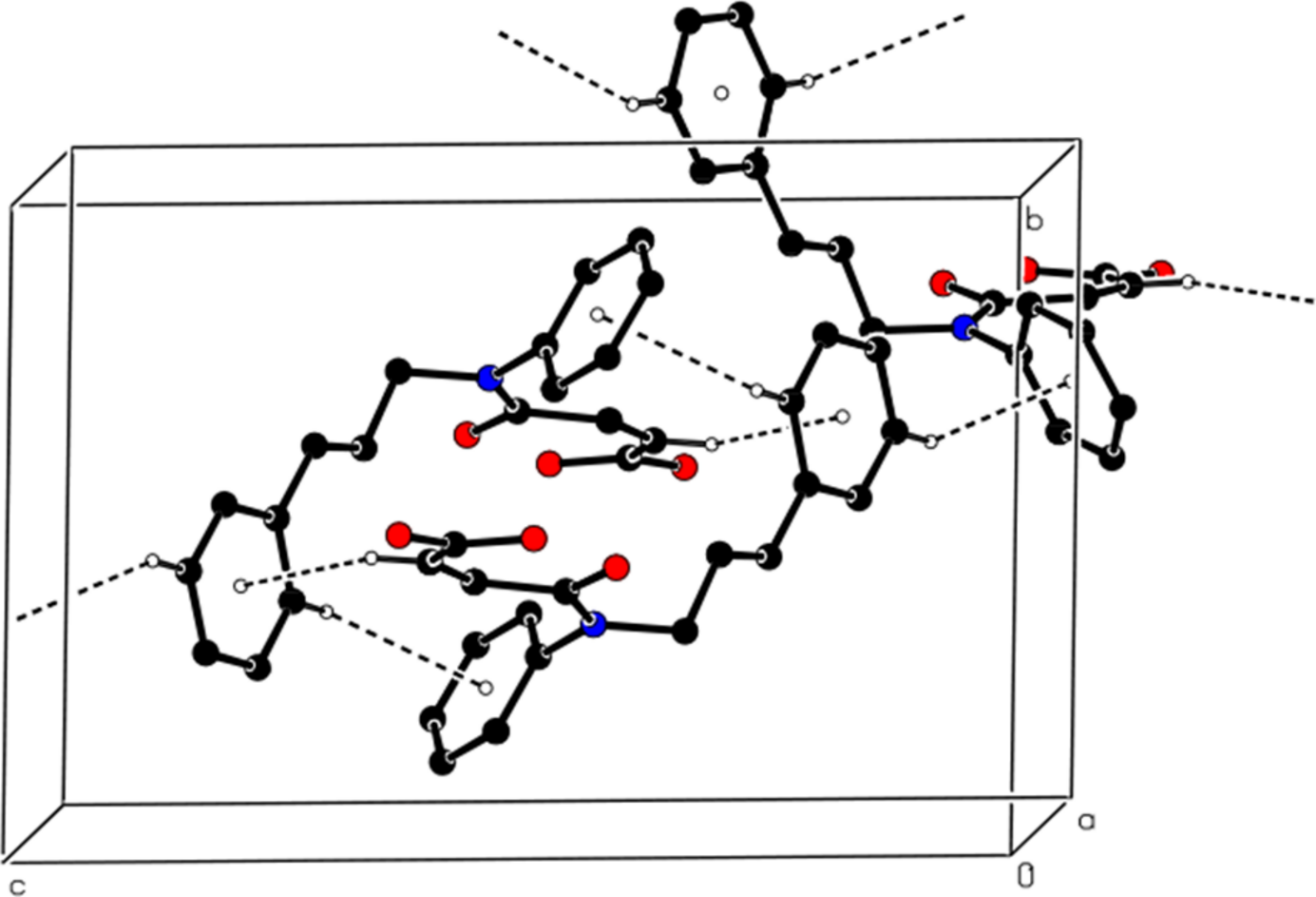
A partial view of the C—H⋯π inter­actions of the title compound in the unit cell. Hydrogen atoms not involved in hydrogen bonding have been omitted for clarity.

In the crystal, mol­ecular pairs are linked by inter­molecular C—H⋯O hydrogen bonds, forming dimers with an 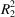
(16) ring motif (Table 1 ▸, Figs. 2 ▸ and 3 ▸). Additionally, C—H⋯π inter­actions connect the mol­ecules into ribbons along the [101] direction (Table 1 ▸, Figs. 4 ▸ and 5 ▸). van der Waals inter­actions between the ribbons consolidate the mol­ecular packing.

**Figure 6:**
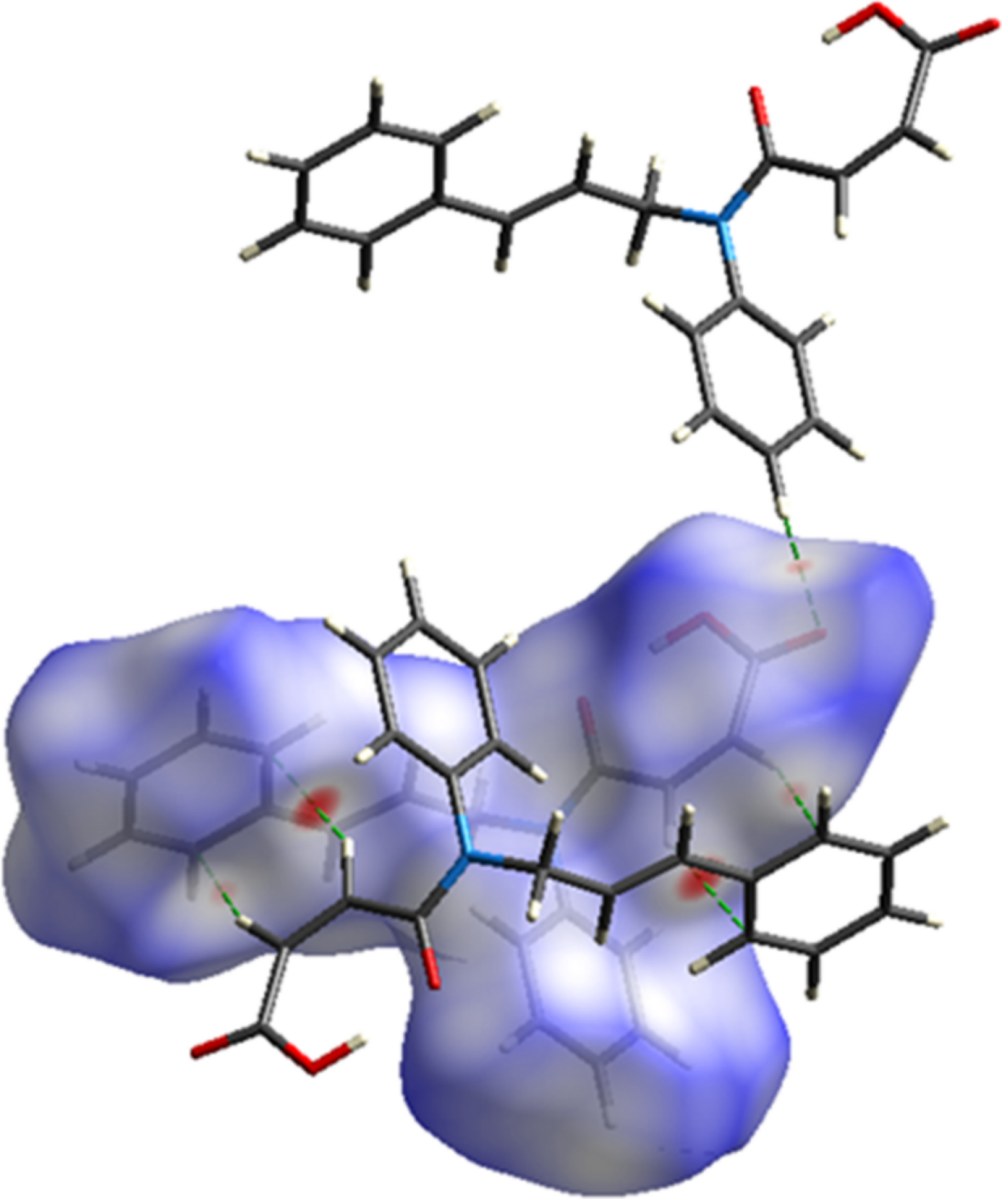
View of the three-dimensional Hirshfeld surface of the title compound plotted over *d*_norm_. The inter­molecular hydrogen bonds are shown with dashed lines.

In order to visualize the inter­molecular inter­actions (Tables 1 ▸ and 2 ▸) in the crystal, a Hirshfeld surface analysis was carried out using *Crystal Explorer 17.5* (Spackman *et al.*, 2021 ▸). Fig. 6 ▸ shows the Hirshfeld surface plotted over *d*_norm_ in the range −0.1614 to 1.4060 a.u. The red spots on the Hirshfeld surface represent O—H⋯O and C—H⋯O contacts.

**Figure 7:**
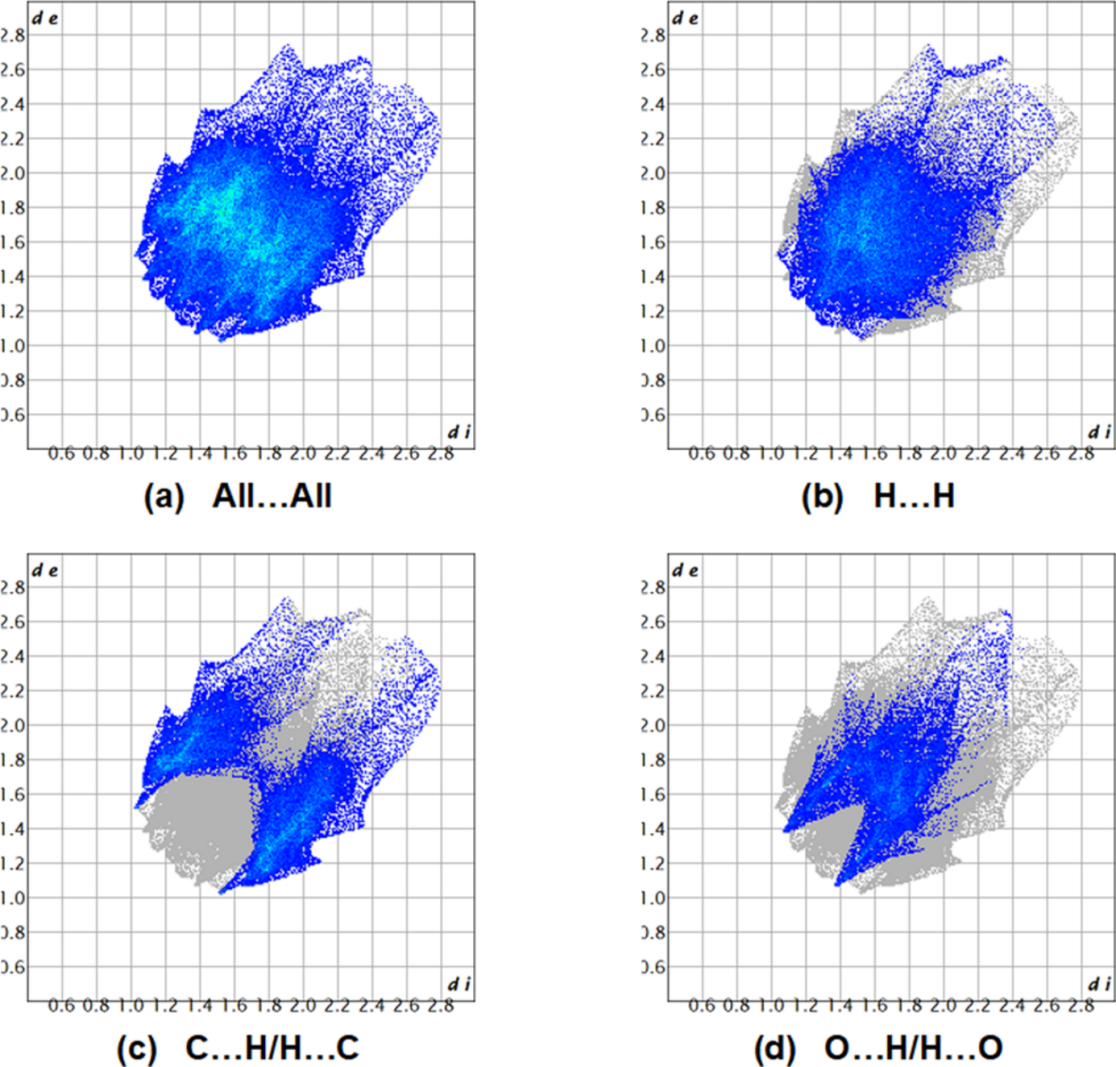
The full two-dimensional fingerprint plots for the title compound, showing (*a*) all inter­actions, and those delineated into (*b*) H⋯H, (*c*) C⋯H/H⋯C and (*d*) O⋯H/H⋯O inter­actions. The *d*_i_ and *d*_e_ values are the closest inter­nal and external distances (in Å) from given points on the Hirshfeld surface.

Fig. 7 ▸ shows the full two-dimensional fingerprint plot and those delineated into the major contacts: H⋯H (Fig. 7 ▸*b*), C⋯H/H⋯C (Fig. 7 ▸*c*) and O⋯H/H⋯O (Fig. 7 ▸*d*) contacts contribute 45.5%, 30.4% and 19.3%, respectively, to the Hirshfeld surface. Specifically, the fingerprint plots reveal the presence of the C⋯H and O⋯H contacts appearing as pairs of spikes with the tips at *d*_e_ + *d*_i_ = 2.53 Å and *d*_e_ + *d*_i_ = 2.40 Å, respectively. The other remaining weak inter­actions (contribution percentages) are O⋯C/C⋯O (3.8%), O⋯O (0.5%), O⋯N/N⋯O (0.2%), N⋯H/H⋯N (0.2%) and C⋯C (0.1%).

## 4. Database survey

A search of the Cambridge Structural Database (CSD, Version 6.00, update of August 2025; Groom *et al.*, 2016 ▸) for the fragment N—C(=O)—C=C—COOH (4-amino-4-oxobut-2-enoic acid) gave in 97 hits. The closely related compounds are CSD refcodes UCOHON (Tahir *et al.*, 2023 ▸), AFIMUA (Dugarte-Dugarte *et al.*, 2019 ▸), IKECUX (Shah *et al.*, 2011 ▸), ANSMAL01 (Gowda *et al.*, 2010*a* ▸), QUYYOZ (Gowda *et al.*, 2010*b* ▸), LOSJUZ (Lo & Ng, 2009 ▸) and BIHXIA (Parvez *et al.*, 2004 ▸).

UCOHON, QUYYOZ and LOSJUZ crystallize in the monoclinic space group *P*2_1_/*c*, with *Z* = 4 for UCOHON and QUYYOZ (*Z* = 8 for LOSJUZ). AFIMUA crystallizes in the monoclinic space group *P*2_1_/*m*, with *Z* = 2. IKECUX crystallizes in the ortho­rhom­bic space group *Pna*2_1_, with *Z* = 4. ANSMAL01 and BIHXIA crystallize in the triclinic space group *P*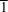, with *Z* = 4.

The torsion angles of the central C—C=C—C group in the N—C(=O)—C=C—COOH fragment are 2.4° for UCOHON, 0.0° for AFIMUA, 1.3° for IKECUX, 0.6° and 3.5° for ANSMAL01 (two mol­ecules in the asymmetric unit), 0.0° for QUYYOZ, 3.7° and 5.2° for LOSJUZ (two mol­ecules in the asymmetric unit), and 1.0° and 0.5° for BIHXIA (two mol­ecules in the asymmetric unit). As can be seen, the torsion angles are smaller than 5.2°, meaning that the functional groups at the ends of the C—C=C—C group point in the same direction and the structures therefore have the same Z configuration.

In UCOHON, mol­ecules are connected as 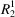
(6) dimers *via* N—H⋯O and C—H⋯O hydrogen bonds. Inter­molecular bonding produces a monoperiodic infinite chain of mol­ecules with a base vector [201]. Furthermore, π-π- stacking enhances the cohesion of the packing. In AFIMUA, mol­ecules are connected by C—H⋯O and N—H⋯O hydrogen bonds, forming layers parallel to the (020) plane. The crystal cohesion is provided by van der Waals inter­actions between the layers. In IKECUX, inter­molecular N—H⋯O bonds lead to the formation of polymer chains propagating along [011]. In ANSMAL01, inter­molecular N—H⋯O hydrogen bonds link the mol­ecules into zigzag chains extending along [1
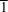
0]. Weak inter­molecular C—H⋯O hydrogen bonds also occur. In QUYYOZ, inter­molecular N—H⋯O hydrogen bonds link the mol­ecules into *C*(7) chains running [010]. In LOSJUZ, adjacent mol­ecules are linked by N—H⋯O hydrogen bonds into a flat ribbon that runs along the [100] direction. In the BIHXIA, the strong inter­molecular N—H⋯O hydrogen bonds create a hydro­phobic area in the centre of the unit cell.

## 5. Synthesis and crystallization

The synthesis of the title compound was described earlier (Voronov *et al.*, 2018 ▸). *N*-[(2*E*)-3-Phenyl­prop-2-en-1-yl]aniline (0.84 g, 4.00 mmol) was dissolved in diethyl ether (5 mL), and maleic anhydride (0.39 g, 4.00 mmol) was added. Hexane (∼4 mL) was then added dropwise until the solution became turbid, after 3–5 drops of diethyl ether were added to restore clarity. The reaction mixture was allowed to stand for 2 h at room temperature. The resulting crystalline precipitate was collected by filtration and dried to afford the target amide as yellowish crystals (1.17 g, 3.80 mmol, 95%, m.p. 362–363 K). The single crystal suitable for XRD analysis was selected from the reaction mixture.

^1^H NMR (600 MHz, CDCl_3_, 294 K) (*J*, Hz): *δ* 7.50–7.20 (*m*, 10 H, H-Ph), 6.48 (*d*, *J* = 16.0, 1 H, H-3-all­yl), 6.27 (*dt*, *J* = 6.6, 16.0, 1 H, H-2-all­yl), 6.20 (*d*, *J* = 13.2, 1 H, H-maleic), 6.16 (*d*, *J* = 13.2, 1 H, H-maleic), 4.55 (*d*, *J* = 6.6, 2 H, H-CH_2_) ppm. ^13^C {^1^H} NMR (151 MHz, CDCl_3_, 294 K) *δ* 165.6, 165.0, 140.0, 135.8 (2C), 135.6, 135.0, 130.1 (2C), 129.2, 128.6, 128.5 (2C), 128.0, 127.4, 126.4 (2C), 121.4, 52.8 ppm. MS (ESI^+^): *m*/*z* (%) = 308.1 [*M* + H]^+^. IR (KBr), ν (cm^−1^) 3420, 3026, 1714, 1625. Analysis calculated for C_19_H_17_NO_3_: C, 74.25; H, 5.58; N, 4.56. Found: C, 74.19; H, 5.32; N, 4.68.

## 6. Refinement

The SC X-ray diffraction data for title compound were collected at the Belok/XSA beamline at the Kurchatov Synchrotron Radiation Source (National Research Center ‘Kurchatov Institute’, Moscow, Russia) at 100 K (Svetogorov *et al.*, 2020 ▸) using a 1-axis MarDTB goniometer equipped with a Rayonix SX165 two-dimensional CCD position-sensitive detector (λ = 0.96990 Å) in direct geometry with the detector plane perpendicular to the photon beam.Crystal data, data collection and structure refinement details are summarized in Table 3 ▸. The OH hydrogen was located in a difference-Fourier map and refined with *U*_iso_(H) = 1.5*U*_eq_(O). The C-bound H-atom positions were calculated geometrically at distances of 0.95 (for aromatic CH) and 0.99 (for CH_2_), and refined using a riding model by applying the constraint *U*_iso_(H) = 1.2*U*_eq_(C). Owing to poor agreement between the observed and calculated intensities, eighteen outliers (0 8 14, −5 5 18, −14 0 20, −15 1 20, 5 5 14, −4 6 17, −13 1 19, −22 2 5, 2 4 16, 1 5 16, −3 9 13, 4 2 15, 1 3 17, −5 9 15, 4 2 16, −2 6 17, 3 7 14 and −12 2 19) were omitted in the final cycles of refinement.

## Supplementary Material

Crystal structure: contains datablock(s) I. DOI: 10.1107/S2056989025010746/vm2320sup1.cif

Structure factors: contains datablock(s) I. DOI: 10.1107/S2056989025010746/vm2320Isup2.hkl

Supporting information file. DOI: 10.1107/S2056989025010746/vm2320Isup3.cml

CCDC reference: 2486561

Additional supporting information:  crystallographic information; 3D view; checkCIF report

Additional supporting information:  crystallographic information; 3D view; checkCIF report

## Figures and Tables

**Figure 3:**
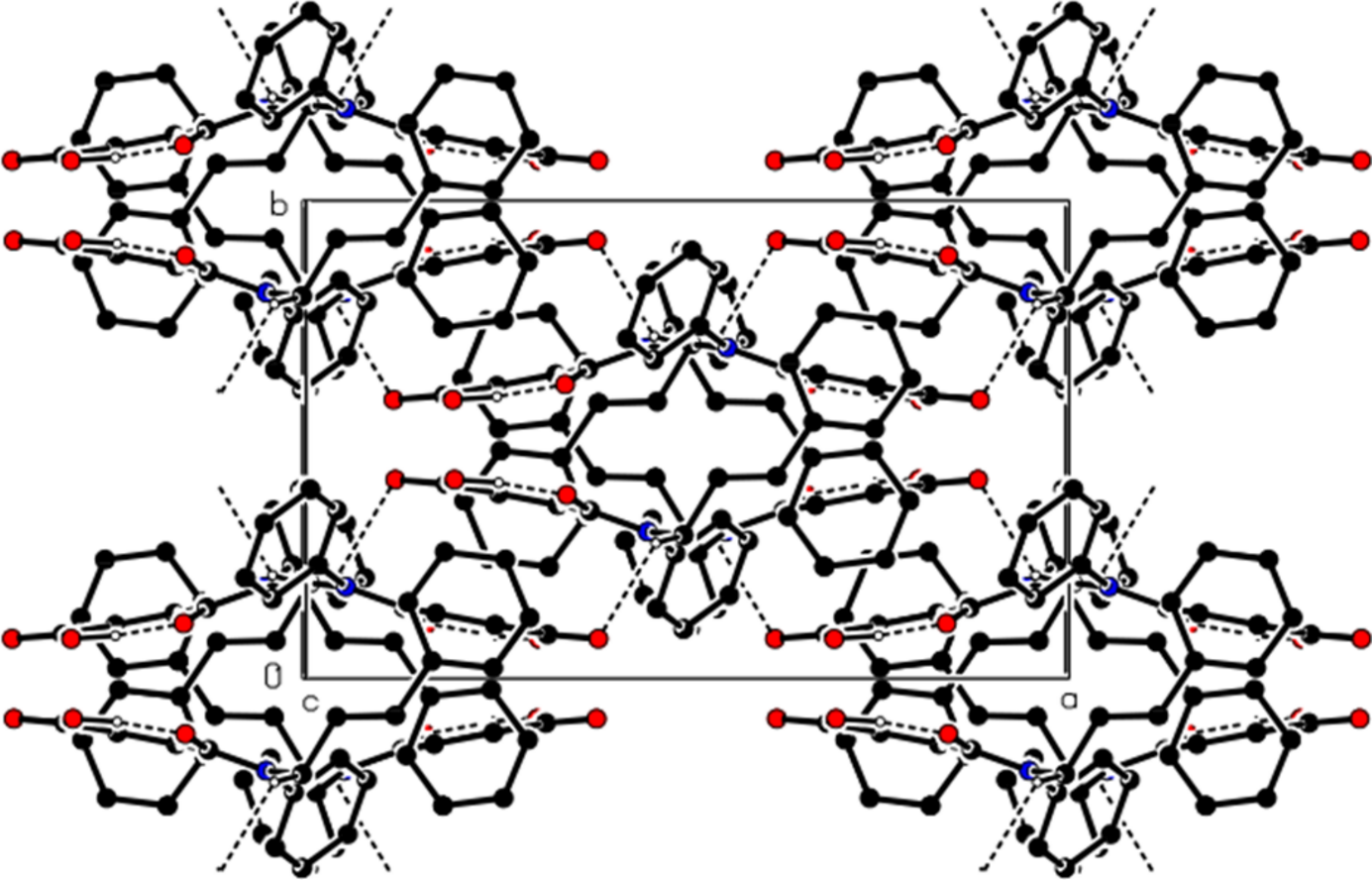
View of the mol­ecular packing of the title compound along the *c* axis. The intra­molecular O—H⋯O and inter­molecular C—H⋯O hydrogen bonds are shown with dashed lines. Hydrogen atoms not involved in hydrogen bonding have been omitted for clarity.

**Figure 5:**
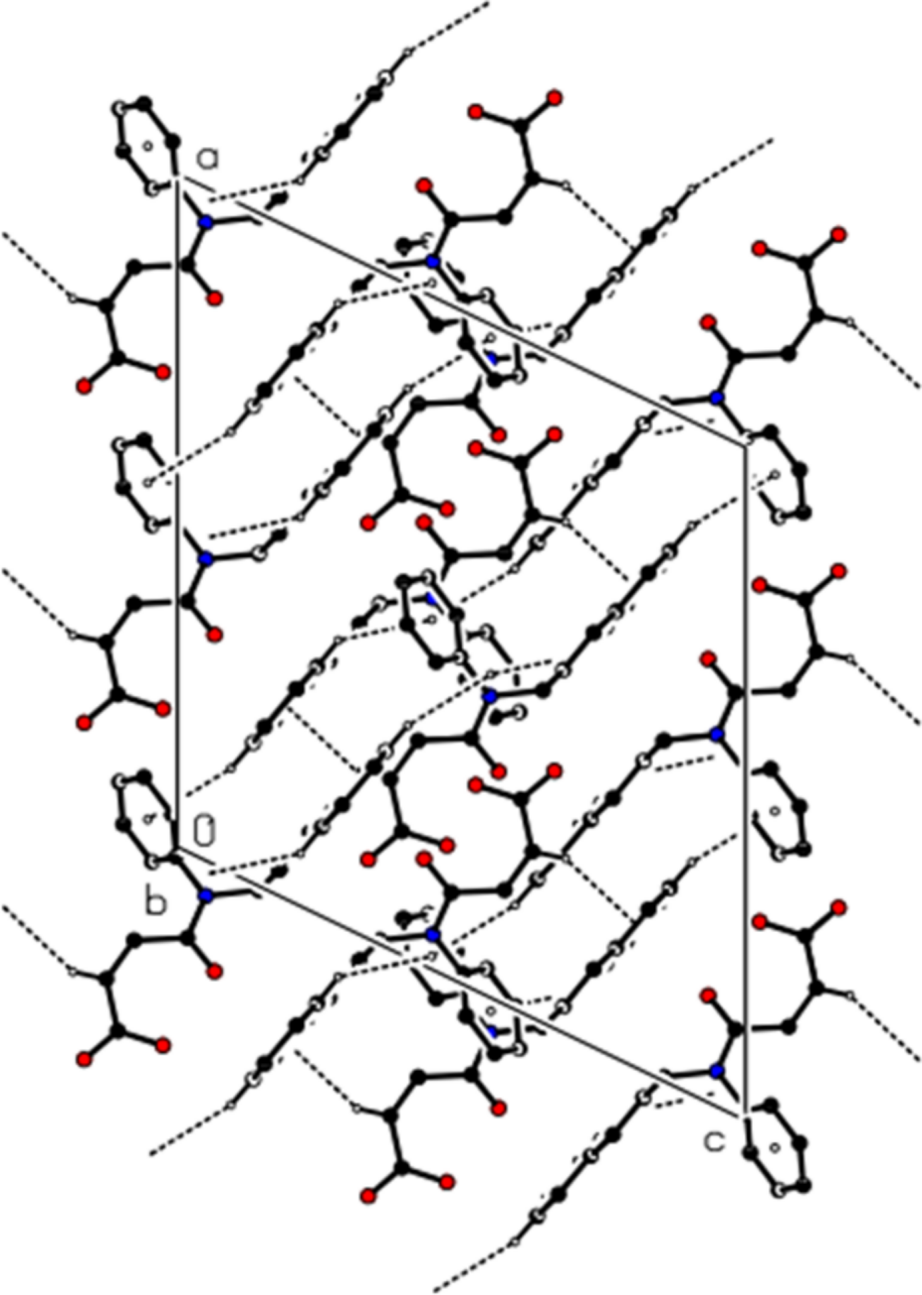
A view of the C—H⋯π inter­actions (dashed lines) of the title compound along the *b* axis. Hydrogen atoms not involved in hydrogen bonding have been omitted for clarity.

**Table 1:** Hydrogen-bond geometry (Å, °) *Cg*1 and *Cg*2 are the centroids of the C8–C13 and C14–C19 phenyl rings, respectively.

*D*—H⋯*A*	*D*—H	H⋯*A*	*D*⋯*A*	*D*—H⋯*A*
C5—H5*B*⋯O1^i^	0.99	2.51	3.2193 (19)	128
O2—H2*O*⋯O3	0.99 (2)	1.54 (2)	2.5341 (19)	173 (2)
C2—H2⋯*Cg*1^ii^	0.95	2.76	3.597 (2)	147
C9—H9⋯*Cg*2^ii^	0.95	2.91	3.6850 (19)	140
C12—H12⋯*Cg*2^iii^	0.95	2.92	3.746 (2)	146

Symmetry codes: (i) 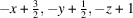; (ii) 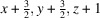; (iii) 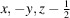.

**Table 2:** Summary of short inter­atomic contacts (Å)

Contact	Distance	Symmetry operation
O1⋯H17	2.62	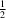 + *x*, 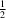 + *y*, *z*
H5*B*⋯O1	2.51	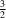 − *x*, 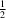 − *y*, 1 − *z*
H5*B*⋯H5*B*	2.56	1 − *x*, *y*, 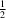 − *z*
H3⋯C9	2.66	1 − *x*, 1 − *y*, 1 − *z*
H10⋯H17	2.56	*x*, 1 − *y*, − 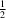 + *z*
C15⋯H11	2.97	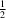 − *x*, − 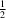 + *y*, 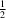 − *z*
H19⋯C18	3.07	1 − *x*, −*y*, 1 − *z*

**Table 3:** Experimental details

Crystal data	
Chemical formula	C_19_H_17_NO_3_
*M*_r_	307.33
Crystal system, space group	Monoclinic, *C*2/*c*
Temperature (K)	100
*a*, *b*, *c* (Å)	18.745 (4), 10.550 (2), 17.540 (4)
β (°)	115.74 (3)
*V* (Å^3^)	3124.5 (13)
*Z*	8
Radiation type	Synchrotron, λ = 0.96990 Å
μ (mm^−1^)	0.20
Crystal size (mm)	0.15 × 0.15 × 0.15

Data collection	
Diffractometer	MAR CCD
Absorption correction	Multi-scan (*SCALA*; Evans, 2006 ▸)
*T*_min_, *T*_max_	0.960, 0.960
No. of measured, independent and observed [*I* > 2σ(*I*)] reflections	25658, 3213, 2779
*R*_int_	0.064
(sin θ/λ)_max_ (Å^−1^)	0.637

Refinement	
*R*[*F*^2^ > 2σ(*F*^2^)], *wR*(*F*^2^), *S*	0.042, 0.132, 1.15
No. of reflections	3213
No. of parameters	212
H-atom treatment	H atoms treated by a mixture of independent and constrained refinement
Δρ_max_, Δρ_min_ (e Å^−3^)	0.25, −0.27

Computer programs: *Automar* (Doyle, 2011 ▸), *iMosflm* (Battye *et al.*, 2011 ▸), *SHELXT* (Sheldrick, 2015*a* ▸), *SHELXL2018* (Sheldrick, 2015*b* ▸), *ORTEP-3 for Windows* (Farrugia, 2012 ▸) and *PLATON* (Spek, 2020 ▸).

## supplementary crystallographic information

### (2Z)-4-Oxo-4-{phenyl[(2E)-3-phenylprop-2-en-1-yl]amino}but-2-enoic acid Crystal data

**Table:**

C_19_H_17_NO_3_	*F*(000) = 1296
*M**_r_* = 307.33	*D*_x_ = 1.307 Mg m^−^^3^
Monoclinic, *C*2/*c*	Synchrotron radiation, λ = 0.96990 Å
*a* = 18.745 (4) Å	Cell parameters from 600 reflections
*b* = 10.550 (2) Å	θ = 3.6–36.0°
*c* = 17.540 (4) Å	µ = 0.20 mm^−^^1^
β = 115.74 (3)°	*T* = 100 K
*V* = 3124.5 (13) Å^3^	Prism, colourless
*Z* = 8	0.15 × 0.15 × 0.15 mm

### (2Z)-4-Oxo-4-{phenyl[(2E)-3-phenylprop-2-en-1-yl]amino}but-2-enoic acid Data collection

**Table:**

MAR CCD diffractometer	2779 reflections with *I* > 2σ(*I*)
/f scan	*R*_int_ = 0.064
Absorption correction: multi-scan (Scala; Evans, 2006)	θ_max_ = 38.2°, θ_min_ = 3.5°
*T*_min_ = 0.960, *T*_max_ = 0.960	*h* = −23→23
25658 measured reflections	*k* = −13→13
3213 independent reflections	*l* = −20→20

### (2Z)-4-Oxo-4-{phenyl[(2E)-3-phenylprop-2-en-1-yl]amino}but-2-enoic acid Refinement

**Table:**

Refinement on *F*^2^	Secondary atom site location: difference Fourier map
Least-squares matrix: full	Hydrogen site location: mixed
*R*[*F*^2^ > 2σ(*F*^2^)] = 0.042	H atoms treated by a mixture of independent and constrained refinement
*wR*(*F*^2^) = 0.132	*w* = 1/[σ^2^(*F*_o_^2^) + (0.0541*P*)^2^ + 3.0894*P*] where *P* = (*F*_o_^2^ + 2*F*_c_^2^)/3
*S* = 1.15	(Δ/σ)_max_ < 0.001
3213 reflections	Δρ_max_ = 0.25 e Å^−^^3^
212 parameters	Δρ_min_ = −0.27 e Å^−^^3^
0 restraints	Extinction correction: SHELXL2019/2 (Sheldrick, 2015b), Fc^*^=kFc[1+0.001xFc^2^λ^3^/sin(2θ)]^-1/4^
Primary atom site location: difference Fourier map	Extinction coefficient: 0.0077 (6)

### (2Z)-4-Oxo-4-{phenyl[(2E)-3-phenylprop-2-en-1-yl]amino}but-2-enoic acid Special details

**Table:**

Geometry. All esds (except the esd in the dihedral angle between two l.s. planes) are estimated using the full covariance matrix. The cell esds are taken into account individually in the estimation of esds in distances, angles and torsion angles; correlations between esds in cell parameters are only used when they are defined by crystal symmetry. An approximate (isotropic) treatment of cell esds is used for estimating esds involving l.s. planes.

### (2Z)-4-Oxo-4-{phenyl[(2E)-3-phenylprop-2-en-1-yl]amino}but-2-enoic acid Fractional atomic coordinates and isotropic or equivalent isotropic displacement parameters (Å^2^)

**Table:**

	*x*	*y*	*z*	*U*_iso_*/*U*_eq_	
C1	0.81635 (9)	0.40744 (13)	0.60505 (11)	0.0235 (4)	
C2	0.74709 (9)	0.38736 (14)	0.62488 (11)	0.0232 (4)	
H2	0.760172	0.392749	0.683533	0.028*	
C3	0.66985 (9)	0.36316 (14)	0.57599 (10)	0.0228 (3)	
H3	0.637932	0.352217	0.605290	0.027*	
C4	0.62796 (9)	0.35132 (13)	0.48221 (10)	0.0207 (3)	
C5	0.50610 (9)	0.30015 (14)	0.35641 (10)	0.0219 (3)	
H5A	0.468419	0.228464	0.342232	0.026*	
H5B	0.542462	0.283513	0.330163	0.026*	
C6	0.46102 (9)	0.42042 (14)	0.32047 (10)	0.0215 (3)	
H6	0.488691	0.498638	0.336577	0.026*	
C7	0.38409 (9)	0.42312 (14)	0.26705 (10)	0.0211 (3)	
H7	0.357350	0.343931	0.253055	0.025*	
C8	0.33638 (9)	0.53712 (14)	0.22753 (10)	0.0204 (3)	
C9	0.36739 (9)	0.66059 (14)	0.24580 (10)	0.0236 (4)	
H9	0.421666	0.672530	0.283325	0.028*	
C10	0.31969 (10)	0.76554 (15)	0.20969 (10)	0.0260 (4)	
H10	0.341277	0.848460	0.223502	0.031*	
C11	0.24038 (10)	0.74943 (15)	0.15334 (11)	0.0284 (4)	
H11	0.207920	0.821191	0.128587	0.034*	
C12	0.20879 (10)	0.62771 (16)	0.13338 (11)	0.0283 (4)	
H12	0.154942	0.616287	0.094289	0.034*	
C13	0.25630 (9)	0.52277 (15)	0.17083 (10)	0.0241 (4)	
H13	0.234092	0.440158	0.157767	0.029*	
C14	0.51582 (8)	0.26228 (14)	0.50220 (10)	0.0205 (3)	
C15	0.45572 (9)	0.33305 (15)	0.50853 (10)	0.0238 (4)	
H15	0.439150	0.411006	0.478917	0.029*	
C16	0.42017 (9)	0.28861 (16)	0.55858 (11)	0.0288 (4)	
H16	0.379095	0.336094	0.563066	0.035*	
C17	0.44493 (10)	0.17476 (16)	0.60189 (12)	0.0312 (4)	
H17	0.420875	0.144754	0.636241	0.037*	
C18	0.50497 (11)	0.10416 (15)	0.59522 (12)	0.0309 (4)	
H18	0.521832	0.026592	0.625254	0.037*	
C19	0.54025 (9)	0.14736 (14)	0.54451 (11)	0.0254 (4)	
H19	0.580441	0.098896	0.538949	0.031*	
N1	0.55259 (7)	0.30759 (11)	0.44989 (8)	0.0202 (3)	
O1	0.88229 (6)	0.41635 (11)	0.66379 (8)	0.0308 (3)	
O2	0.80530 (7)	0.41655 (11)	0.52536 (8)	0.0287 (3)	
H2O	0.7476 (14)	0.410 (2)	0.4880 (14)	0.043*	
O3	0.65846 (6)	0.38441 (11)	0.43448 (7)	0.0257 (3)	

### (2Z)-4-Oxo-4-{phenyl[(2E)-3-phenylprop-2-en-1-yl]amino}but-2-enoic acid Atomic displacement parameters (Å^2^)

**Table:**

	*U*^11^	*U*^22^	*U*^33^	*U*^12^	*U*^13^	*U*^23^
C1	0.0237 (8)	0.0162 (7)	0.0309 (10)	0.0003 (5)	0.0121 (7)	−0.0005 (6)
C2	0.0239 (8)	0.0209 (7)	0.0233 (9)	0.0002 (6)	0.0087 (6)	0.0000 (6)
C3	0.0238 (7)	0.0219 (7)	0.0248 (9)	−0.0006 (6)	0.0124 (6)	−0.0007 (6)
C4	0.0207 (7)	0.0174 (7)	0.0236 (8)	0.0018 (5)	0.0093 (6)	0.0008 (6)
C5	0.0224 (7)	0.0227 (7)	0.0185 (8)	0.0027 (6)	0.0070 (6)	−0.0005 (6)
C6	0.0249 (7)	0.0195 (7)	0.0201 (8)	0.0008 (5)	0.0100 (6)	0.0004 (6)
C7	0.0225 (7)	0.0204 (7)	0.0226 (8)	−0.0003 (5)	0.0118 (6)	−0.0021 (6)
C8	0.0219 (7)	0.0216 (7)	0.0194 (8)	0.0019 (5)	0.0106 (6)	0.0001 (6)
C9	0.0214 (7)	0.0230 (7)	0.0270 (9)	−0.0004 (6)	0.0111 (6)	0.0002 (6)
C10	0.0308 (8)	0.0204 (7)	0.0281 (9)	0.0004 (6)	0.0141 (7)	0.0000 (6)
C11	0.0296 (8)	0.0242 (8)	0.0297 (9)	0.0084 (6)	0.0113 (7)	0.0043 (7)
C12	0.0233 (8)	0.0301 (8)	0.0273 (9)	0.0038 (6)	0.0071 (7)	−0.0006 (7)
C13	0.0237 (7)	0.0230 (7)	0.0240 (9)	0.0000 (6)	0.0089 (6)	−0.0025 (6)
C14	0.0191 (7)	0.0202 (7)	0.0206 (8)	−0.0035 (5)	0.0072 (6)	−0.0025 (6)
C15	0.0196 (7)	0.0226 (7)	0.0262 (9)	−0.0014 (6)	0.0074 (6)	−0.0052 (6)
C16	0.0233 (8)	0.0313 (8)	0.0344 (10)	−0.0063 (6)	0.0149 (7)	−0.0102 (7)
C17	0.0353 (9)	0.0302 (8)	0.0340 (10)	−0.0152 (7)	0.0205 (8)	−0.0093 (7)
C18	0.0416 (10)	0.0208 (7)	0.0324 (10)	−0.0064 (6)	0.0179 (8)	−0.0022 (7)
C19	0.0269 (8)	0.0193 (7)	0.0313 (9)	−0.0009 (6)	0.0138 (7)	−0.0022 (6)
N1	0.0195 (6)	0.0199 (6)	0.0200 (7)	0.0011 (5)	0.0074 (5)	0.0005 (5)
O1	0.0208 (6)	0.0285 (6)	0.0381 (8)	−0.0001 (4)	0.0082 (5)	−0.0003 (5)
O2	0.0250 (6)	0.0324 (6)	0.0319 (7)	0.0007 (5)	0.0153 (5)	0.0043 (5)
O3	0.0274 (6)	0.0291 (6)	0.0233 (6)	−0.0027 (4)	0.0135 (5)	0.0027 (5)

### (2Z)-4-Oxo-4-{phenyl[(2E)-3-phenylprop-2-en-1-yl]amino}but-2-enoic acid Geometric parameters (Å, º)

**Table:**

C1—O1	1.221 (2)	C10—C11	1.393 (2)
C1—O2	1.325 (2)	C10—H10	0.9500
C1—C2	1.498 (2)	C11—C12	1.394 (2)
C2—C3	1.348 (2)	C11—H11	0.9500
C2—H2	0.9500	C12—C13	1.394 (2)
C3—C4	1.489 (2)	C12—H12	0.9500
C3—H3	0.9500	C13—H13	0.9500
C4—O3	1.2511 (19)	C14—C19	1.391 (2)
C4—N1	1.3544 (19)	C14—C15	1.395 (2)
C5—N1	1.488 (2)	C14—N1	1.447 (2)
C5—C6	1.503 (2)	C15—C16	1.394 (2)
C5—H5A	0.9900	C15—H15	0.9500
C5—H5B	0.9900	C16—C17	1.389 (3)
C6—C7	1.336 (2)	C16—H16	0.9500
C6—H6	0.9500	C17—C18	1.396 (3)
C7—C8	1.479 (2)	C17—H17	0.9500
C7—H7	0.9500	C18—C19	1.395 (2)
C8—C13	1.403 (2)	C18—H18	0.9500
C8—C9	1.406 (2)	C19—H19	0.9500
C9—C10	1.391 (2)	O2—H2O	0.99 (2)
C9—H9	0.9500		
			
O1—C1—O2	121.44 (15)	C11—C10—H10	119.9
O1—C1—C2	118.47 (15)	C10—C11—C12	119.79 (14)
O2—C1—C2	120.09 (14)	C10—C11—H11	120.1
C3—C2—C1	132.74 (16)	C12—C11—H11	120.1
C3—C2—H2	113.6	C13—C12—C11	119.92 (15)
C1—C2—H2	113.6	C13—C12—H12	120.0
C2—C3—C4	128.61 (15)	C11—C12—H12	120.0
C2—C3—H3	115.7	C12—C13—C8	121.08 (14)
C4—C3—H3	115.7	C12—C13—H13	119.5
O3—C4—N1	120.78 (15)	C8—C13—H13	119.5
O3—C4—C3	122.75 (14)	C19—C14—C15	120.92 (15)
N1—C4—C3	116.41 (14)	C19—C14—N1	119.27 (13)
N1—C5—C6	111.80 (12)	C15—C14—N1	119.80 (13)
N1—C5—H5A	109.3	C16—C15—C14	119.57 (15)
C6—C5—H5A	109.3	C16—C15—H15	120.2
N1—C5—H5B	109.3	C14—C15—H15	120.2
C6—C5—H5B	109.3	C17—C16—C15	119.85 (15)
H5A—C5—H5B	107.9	C17—C16—H16	120.1
C7—C6—C5	123.45 (14)	C15—C16—H16	120.1
C7—C6—H6	118.3	C16—C17—C18	120.37 (16)
C5—C6—H6	118.3	C16—C17—H17	119.8
C6—C7—C8	126.49 (14)	C18—C17—H17	119.8
C6—C7—H7	116.8	C19—C18—C17	120.10 (16)
C8—C7—H7	116.8	C19—C18—H18	119.9
C13—C8—C9	118.09 (13)	C17—C18—H18	119.9
C13—C8—C7	119.17 (13)	C14—C19—C18	119.17 (15)
C9—C8—C7	122.73 (13)	C14—C19—H19	120.4
C10—C9—C8	120.90 (14)	C18—C19—H19	120.4
C10—C9—H9	119.6	C4—N1—C14	123.00 (13)
C8—C9—H9	119.6	C4—N1—C5	118.99 (13)
C9—C10—C11	120.20 (15)	C14—N1—C5	117.96 (12)
C9—C10—H10	119.9	C1—O2—H2O	108.4 (13)
			
O1—C1—C2—C3	−173.25 (16)	N1—C14—C15—C16	179.61 (14)
O2—C1—C2—C3	7.5 (3)	C14—C15—C16—C17	0.2 (2)
C1—C2—C3—C4	−1.5 (3)	C15—C16—C17—C18	−0.3 (2)
C2—C3—C4—O3	−12.6 (2)	C16—C17—C18—C19	−0.3 (3)
C2—C3—C4—N1	170.26 (15)	C15—C14—C19—C18	−1.3 (2)
N1—C5—C6—C7	−130.63 (16)	N1—C14—C19—C18	179.70 (14)
C5—C6—C7—C8	−178.36 (14)	C17—C18—C19—C14	1.2 (2)
C6—C7—C8—C13	178.36 (16)	O3—C4—N1—C14	176.87 (13)
C6—C7—C8—C9	−3.2 (3)	C3—C4—N1—C14	−6.0 (2)
C13—C8—C9—C10	1.0 (2)	O3—C4—N1—C5	−0.4 (2)
C7—C8—C9—C10	−177.52 (15)	C3—C4—N1—C5	176.76 (12)
C8—C9—C10—C11	−1.2 (3)	C19—C14—N1—C4	−71.30 (19)
C9—C10—C11—C12	0.2 (3)	C15—C14—N1—C4	109.69 (16)
C10—C11—C12—C13	1.0 (3)	C19—C14—N1—C5	105.99 (16)
C11—C12—C13—C8	−1.2 (3)	C15—C14—N1—C5	−73.02 (17)
C9—C8—C13—C12	0.2 (2)	C6—C5—N1—C4	−90.16 (16)
C7—C8—C13—C12	178.78 (15)	C6—C5—N1—C14	92.43 (15)
C19—C14—C15—C16	0.6 (2)		

### (2Z)-4-Oxo-4-{phenyl[(2E)-3-phenylprop-2-en-1-yl]amino}but-2-enoic acid Hydrogen-bond geometry (Å, º)

*Cg*1 and *Cg*2 are the centroids of the C8–C13 and C14–C19 phenyl rings, respectively.

**Table:**

*D*—H···*A*	*D*—H	H···*A*	*D*···*A*	*D*—H···*A*
C5—H5*B*···O1^i^	0.99	2.51	3.2193 (19)	128
O2—H2*O*···O3	0.99 (2)	1.54 (2)	2.5341 (19)	173 (2)
C2—H2···*Cg*1^ii^	0.95	2.76	3.597 (2)	147
C9—H9···*Cg*2^ii^	0.95	2.91	3.6850 (19)	140
C12—H12···*Cg*2^iii^	0.95	2.92	3.746 (2)	146

Symmetry codes: (i) −*x*+3/2, −*y*+1/2, −*z*+1; (ii) *x*+3/2, *y*+3/2, *z*+1; (iii) *x*, −*y*, *z*−1/2.
